# Clinical and Epidemiological Spectrum of Acute Viral Hepatitis Due to Hepatitis A and E in Children: A Descriptive, Cross-Sectional, Hospital-Based Study

**DOI:** 10.7759/cureus.24056

**Published:** 2022-04-12

**Authors:** Javaria Rasheed, Muhammad Khalid, Sobia Rubab, Bushra Iqbal, Iram Nawaz, Asad Shahzad

**Affiliations:** 1 Pediatrics, Nishtar Medical University, Multan, PAK; 2 Pediatrics, Bakhtawar Amin Medical and Dental College, Multan, PAK

**Keywords:** liver function tests, enzyme-linked immunosorbent assay, viral hepatitis, hepatitis e, hepatitis a, child

## Abstract

Objective: Acute viral hepatitis (AVH) in children is a serious and major public health concern globally and in developing countries such as Pakistan. We conducted this study to determine the clinical and epidemiological spectrum of AVH due to hepatitis A virus (HAV) and hepatitis E virus (HEV) infection in children.

Methodology: This cross-sectional study was conducted at the Pediatric Medicine Department of a tertiary care hospital from February 20, 2020, to February 20, 2022. A total of 200 children 1-12 years of age who presented with symptoms and signs of AVH were enrolled. Demographic and clinical characteristics were noted, and venous blood was drawn for the assessment of HAV IgM and HEV IgM using an enzyme-linked immunosorbent assay (ELISA). Descriptive statistics are run, and the results are presented as tables.

Results: Of the children, 75% were diagnosed with acute HAV infection. The median duration of illness was six days (range: 2-21 days). The most common age group affected was 6-10 years (43.5%), of which 56.5% were males. Most of the children belonged to low and middle socioeconomic status (86.5%), and 41.5% consumed underground water for drinking. Fever was the most common symptom, followed by appetite loss and yellow discoloration of urine. Alanine aminotransferase (ALT) was significantly high in HEV compared to HAV infection (2060.2±1036.7 versus 1730.7±957.5 IU/L) (P=0.04).

Conclusion: Acute HAV was more prevalent. Those who are male, 6-10 years of age, from lower and middle socioeconomic status, and using underground drinking water were more affected by acute viral hepatitis. The clinical and biochemical presentation of HAV and HEV did not differ significantly.

## Introduction

Hepatitis A virus (HAV) and hepatitis E virus (HEV) are hepatotropic viruses and among the most common feco-orally transmitted pathogens leading to acute viral hepatitis (AVH) [1]. AVH is an important health problem among children in the developing world, especially in the rural and slum areas of lower socioeconomic status. People are affected due to a lack of safe drinking water and poor sanitation and personal hygiene [2]. According to the WHO estimates, every year, 1.4 million new cases of HAV and 20 million cases of HEV are reported globally. Approximately 100,000 and 60,000 people/year die because of acute HAV and HEV infection, respectively. It is also estimated that, by 10 years of age, 90% of children are infected with hepatitis A [3,4]. In the Indian subcontinent, the proportion of overall acute viral hepatitis, acute liver failure, and acute-on-chronic liver failure cases attributed to HAV infection is around 70%-85%, 40%-60%, and 10%-40%, respectively [5].

Hepatitis A virus and hepatitis E virus infections occur both in outbreaks and sporadically. The spread of infection has been documented both in domestic and institutional settings. The common implicated factors are consumption of contaminated food, water, milk, or dairy products propagated by poor sanitation and overcrowding. Both HAV and HEV infections are self-limiting and provide lifelong immunity [6]. Children with AVH most commonly present with nonspecific gastrointestinal symptoms in addition to fever. The visible yellowness of the eyes and urine (due to high bilirubin) is followed by abdominal pain, vomiting, nausea, and appetite loss with variable frequency. Rare manifestations include fulminant hepatic failure, aplastic anemia, and prolonged cholestatic syndrome [7,8].

Routine vaccination for HAV is not advocated by the Expanded Program of Immunization in Pakistan. This is likely due to insufficient seroprevalence data, particularly for pediatric population. The assessment of the need to vaccinate the high-risk groups (such as pregnant women, those with chronic liver disease, and young children) is essential to make for HAV vaccination and preventive strategies for HEV infection in children in countries such as Pakistan. Against this background, we conducted this study in our hospital to understand the epidemiology and clinical presentations of acute HAV and HEV infection in children.

## Materials and methods

This hospital-based, cross-sectional study was carried out from February 20, 2020, to February 20, 2022, at the Department of Pediatric Medicine of Nishtar Medical University, Multan, Pakistan. The study was approved by the Institutional Ethical Review Board (IERB) (#4000/NMU & H) on February 19, 2020. Children 1-12 years of age who presented with acute hepatitis symptoms and signs were included in the study through consecutive sampling after informed consent was obtained from the parents. Children with a history of preexisting liver disease were excluded from the study. A case of acute hepatitis was defined as a child presenting with acute illness of ≤21 days duration with any sign or symptom (e.g., fever, yellowness of the eyes and urine, loss of appetite, nausea, vomiting, and abdominal pain) and elevated alanine aminotransferase (ALT).

A structured proforma was used to collect data from each case. Demographic characteristics, including age, gender, parental education, area of residence, socioeconomic status, number of children in each household, source of drinking water, and duration of illness, were recorded. Socioeconomic status was assessed using the World Bank classification based on monthly household income [9]. It was categorized as low (≤11,605 Pakistani rupee (PKR)), lower-middle (11,606-45,395 PKR), upper-middle (45,396-140,461 PKR), and high (>140,461 PKR). Clinical presentations (fever, vomiting, nausea, yellowness of the eyes and urine, pain abdomen, and appetite loss) were noted. Through the aseptic technique, 5 mL of venous blood was collected from all children for the assessment of anti-HAV and anti-HEV IgM antibodies. Serum was separated from the collected blood samples after centrifugation at 1000 revolutions per minute (rpm) for five minutes. According to the manufacturer’s guide, IgM for HAV and HEV were analyzed using a commercially available enzyme-linked immunosorbent assay (ELISA) test (DiaPro Diagnostic Bioprobes, MI, Italy). Other laboratory parameters done as panel for acute hepatitis were prothrombin time (PT), international normalized ratio (INR), ALT, aspartate aminotransferase (AST), alkaline phosphatase (ALP), and total bilirubin.

A minimum sample size of 189 children was calculated taking prevalence of HAV as 60% [10], confidence level of 95%, and 7% margin of error. The SPSS software version 24 for Windows (IBM Corporation, Armonk, NY, USA) was used for data analysis. Descriptive statistics in the form of mean±SD and frequency (%) are utilized for numerical and categorical variables, respectively. Chi-square test and independent sample t-test were applied for the comparison of categorical and numerical data between HAV and HEV groups. P-value ≤ 0.05 was taken as significant.

## Results

Of 200 children with acute hepatitis, 75% (n=150) were diagnosed with acute hepatitis A and 25% (n=50) with acute hepatitis E. The mean age of the participants was 7.3±3.0 years, and most (43.5%) belonged to the 6-10 years age group. There were 113 (56.5%) males, and 51.5% (n=103) came from urban areas. There were 28.5%, 36.5%, 21.5%, and 13.5% children representing low, lower-middle, upper-middle, and high socioeconomic status, respectively. Only 15.5% of mothers had education status as graduate and above, and 26.5%, 29.5%, 22%, and 16.5% were in the illiterate, primary, secondary, and higher secondary education categories. The median number of children in a household was three (range: 1-8), and the median duration of symptoms was six days (range: 2-21 days). The most common source of drinking water in the household was underground boring (41.5%, n=83), followed by municipal water supply (34%, n=68) and bottled water (24.5%, n=9). The characteristics of children diagnosed with HAV and HEV infection did not differ significantly (P>0.05) (Table 1).

**Table 1:** Characteristics of children presenting with acute viral hepatitis (N=200) * Chi-square test ^¥^ Mann-Whitney U test ^€^ Independent sample t-test

Characteristics	Total # of children (%)	# of children with HAV (%)	# of children with HEV (%)	P
Age (years)	7.3±3.0	7.3±3.0	7.2±3.0	0.92^€^
≤5	73 (36.5)	54 (36)	19 (38)	0.92*
6-10	87 (43.5)	65 (43.4)	22 (44)	
>10	40 (20)	31 (20.6)	9 (18)	
Gender				
Male	113 (56.5)	83 (55.3)	30 (60)	0.56*
Female	87 (43.5)	67 (44.7)	20 (40)	
Area of residence				
Rural	97 (48.5)	75 (50)	22 (44)	0.46*
Urban	103 (51.5)	75 (50)	28 (56)	
Socioeconomic status				
Low	57 (28.5)	37 (24.7)	20 (40)	0.21*
Lower-middle	73 (36.5)	57 (38)	16 (32)	
Upper-middle	43 (21.5)	35 (23.3)	8 (16)	
High	27 (13.5)	21 (14)	6 (12)	
Maternal education				
Illiterate	53 (26.5)	42 (28)	11 (22)	0.19*
Primary education	39 (19.5)	27 (18)	12 (24)	
Secondary education	44 (22)	32 (21.3)	12 (24)	
Higher secondary education	33 (16.5)	29 (19.3)	4 (8)	
Graduate and above	31 (15.5)	20 (13.3)	11 (22)	
# of children in house	3 (1-8)	3 (1-8)	3 (1-5)	0.58^¥^
Source of drinking water				
Municipal supply	68 (34)	45 (30)	23 (46)	0.08*
Bottled water	49 (24.5)	37 (24.7)	12 (24)	
Underground water	83 (41.5)	68 (45.3)	15 (30)	
Duration of illness (days)	6 (2-21)	6.5 (2-21)	6 (3-20)	0.74^¥^

The common presenting complaints were fever and appetite loss (71%, n=142, for both). The dark color of the urine was the only complaint reported to be significantly higher in HAV-positive patients compared to HEV-positive patients (P=0.001) (Table 2).

**Table 2:** Frequency of presenting complaints in children with acute viral hepatitis (N=200)

Presenting complaint	Overall (N=200)	HAV (n=150)	HEV (n=50)	P
Fever	142 (71)	110 (73.3)	32 (64)	0.21
Vomiting	114 (57)	81 (54)	33 (66)	0.14
Abdominal pain	91 (45.5)	69 (46)	22 (44)	0.81
Yellowness of the eyes	134 (67)	100 (66.7)	34 (68)	0.86
Dark-colored urine	131 (65.5)	108 (72)	23 (46)	0.001
Nausea	88 (44)	68 (45.3)	20 (40)	0.51
Loss of appetite	142 (71)	106 (70.7)	36 (72)	0.86

The mean serum bilirubin level was 6.0±2.2 mg/dL. The average ALT, AST, and ALP levels were 1813.1±985.7, 1762.7±1000.6, and 630.3±264.2 IU/L, respectively. The mean international normalized ratio (INR) was 1.9±0.6. Only ALT levels were significantly high in HEV patients compared to HAV patients (2060.2±1036.7 versus 1730.7±957.5) (P=0.04). The remaining biochemical parameters did not differ significantly between patients with HAV and HEV infection (P>0.05) (Table 3).

**Table 3:** Biochemical parameters in children presenting with acute viral hepatitis (N=200) ALT: alanine aminotransferase, AST: aspartate aminotransferase, ALP: alkaline phosphatase, INR: international normalized ratio

Parameter	Overall (N=200)	HAV (n=150)	HEV (n=50)	P
Bilirubin (mg/dL)	6.0±2.2	6.0±2.3	6.2±2.1	0.70
ALT (IU/L)	1813.1±985.7	1730.7±957.5	2060.2±1036.7	0.04
AST (IU/L)	1762.7±1000.6	1738.9±998.2	1834.2±1014.6	0.56
ALP (IU/L)	630.3±264.2	641.0±269.1	597.9±248.8	0.32
INR	1.9±0.6	1.8±0.6	1.9±0.6	0.49

## Discussion

We found HAV infection in three-fourths of the study participants as the more common cause of acute hepatitis compared to HEV infection. The results are in line with those documented by Das et al., where HAV was present in 73.2% and HEV was present in 10.7% of cases [11]. The results are also comparable with the results reported by Behera and Patnaik [12]. They found HAV (63.15%) infection as the most common cause of acute viral hepatitis in children, followed by HBV (10.52%), and then HEV (5.26%). Semwal et al. also observed that the frequency of HAV was 95.08% and that of HEV was 13.11% [13]. The prevalence of HAV has reduced in developing countries due to the availability of its vaccination, but it still remains high in countries without routine vaccination against HAV [14].

Male children dominated our study cohort. A study from Karachi included 360 patients with acute hepatitis, and it was found to be dominated by males (86%) as well [15]. Studies from India have also demonstrated that males are more affected by acute hepatitis compared to females [12,16]. Rana and Lone also observed a high prevalence of HAV in male patients (60.8%) compared to female patients (39.13%) [17]. Infections with viruses are more prevalent in human males than in females, suggesting that behavioral or occupational exposures contribute to the acquisition of infection or health-seeking behavior in the community [18].

In our study, the majority of cases belonged to the 6-10 years age group. A similar age group was more affected by acute hepatitis in the study by Sharma et al. [19]. In a study from Bangladesh, 52% of the affected children were in the 5-10 years age group [20]. The consumption of food and water from open hotels and fast-food restaurants in the crowded slum areas with poor hygienic conditions puts this age group at high risk of getting infections [21].

Our study showed that a significant percentage of mothers were either illiterate or had acquired only a primary level of education, while the commonest source of drinking water in the household was underground boring. Pereira et al. observed that the education level of individuals was independently associated with acute hepatitis infection [22]. Ashraf et al. reported an outbreak of hepatitis E in Islamabad, Pakistan. The main reason was open sewage drains located adjacent to water boring pumps, which were at a lower surface level [23]. In another study from Lahore, Pakistan, the authors found that, among children infected with acute hepatitis, the common drinking water sources were local bore water and water fetched from filtration plants [24].

In this study, the most common presenting complaint was fever, followed by appetite loss and yellow discoloration of urine. However, yellow discoloration of urine was the only complaint reported to be significantly higher in HAV-positive patients compared to HEV-positive patients. In a study by Das et al. [11], icterus (100%) was the most common presenting symptom, followed by fever (96.42%), and dark-colored urine (83.92%). Girish et al. also found icterus (100%) as the commonest symptom [25].

## Conclusions

Acute viral hepatitis due to HAV was more prevalent. The commonest age group was 6-10 years. Vaccination against hepatitis A should be part of routine immunization program. Health education should be provided to the general public regarding preventive measures including the use of safe drinking water, proper hand hygiene, proper waste disposal, and the use of sanitary latrines. Awareness of the clinical symptoms of acute viral hepatitis is also important for the early identification and seeking of medical attention so as to prevent morbidity and mortality related to the disease.
